# Characterization of ductal and lobular breast carcinomas using novel prolactin receptor isoform specific antibodies

**DOI:** 10.1186/1471-2407-10-678

**Published:** 2010-12-13

**Authors:** Erika Ginsburg, Stefanie Alexander, Sarah Lieber, Sarah Tarplin, Luwanda Jenkins, Linda Pang, Christopher D Heger, Paul Goldsmith, Barbara K Vonderhaar

**Affiliations:** 1 Mammary Biology and Tumorigenesis Laboratory, National Cancer Institute, National Institutes of Health, Bethesda, MD 20892 USA; 2 Antibody and Protein Purification Unit, National Cancer Institute, National Institutes of Health, Bethesda, MD 20892 USA

## Abstract

**Background:**

Prolactin is a polypeptide hormone responsible for proliferation and differentiation of the mammary gland. More recently, prolactin's role in mammary carcinogenesis has been studied with greater interest. Studies from our laboratory and from others have demonstrated that three specific isoforms of the prolactin receptor (PRLR) are expressed in both normal and cancerous breast cells and tissues. Until now, reliable isoform specific antibodies have been lacking. We have prepared and characterized polyclonal antibodies against each of the human PRLR isoforms that can effectively be used to characterize human breast cancers.

**Methods:**

Rabbits were immunized with synthetic peptides of isoform unique regions and immune sera affinity purified prior to validation by Western blot and immunohistochemical analyses. Sections of ductal and lobular carcinomas were stained with each affinity purified isoform specific antibody to determine expression patterns in breast cancer subclasses.

**Results:**

We show that the rabbit antibodies have high titer and could specifically recognize each isoform of PRLR. Differences in PRLR isoform expression levels were observed and quantified using histosections from xenografts of established human breast cancer cells lines, and ductal and lobular carcinoma human biopsy specimens. In addition, these results were verified by real-time PCR with isoform specific primers. While nearly all tumors contained LF and SF1b, the majority (76%) of ductal carcinoma biopsies expressed SF1a while the majority of lobular carcinomas lacked SF1a staining (72%) and 27% had only low levels of expression.

**Conclusions:**

Differences in the receptor isoform expression profiles may be critical to understanding the role of PRL in mammary tumorigenesis. Since these antibodies are specifically directed against each PRLR isoform, they are valuable tools for the evaluation of breast cancer PRLR content and have potential clinical importance in treatment of this disease by providing new reagents to study the protein expression of the human PRLR.

## Background

The role of prolactin (PRL) in human breast cancer is now becoming more clearly defined. Recent epidemiologic evidence clearly shows that in both pre- and post-menopausal women with serum prolactin levels in the highest quartile have a significant increased risk of developing breast cancer [1,2]. PRL, acting through is receptors, has definitively been shown to increase cell proliferation and decrease apoptosis in breast cancer cells in culture [3,4]. Additionally, PRL is a pro-angiogenesis factor both in normal and cancerous mammary tissue [5,6]. We [7] and others [8] have shown the existence of several receptor isoforms whose involvement in PRL-induced cell proliferation and decreased apoptosis remains to be fully defined.

The PRLR is a member of the class I cytokine/hematopoietic receptor superfamily, characterized by a single hydrophobic transmembrane region that separates the ligand-binding extracellular from the signaling intracellular domain. There are five cell-associated isoforms of the human PRLR, long (LF), intermediate, ΔS1, and two short forms (SF1a and SF1b) [4,9] that differ only in their C-terminal cytoplasmic domains. The expression of the PRLR is regulated by PRL itself where low levels of PRL upregulate and high levels of PRL downregulate the receptor [10]. The three major cell associated isoforms of the PRLR, the LF, that signals for all known functions including growth and differentiation, and two short forms, SF1a and SF1b, whose functions, other than their ability to act as dominant negatives for differentiation in transfected cultured cells [7,8,11,12], are still largely undefined. Studies from our laboratory and from others [7,12] have demonstrated that mRNA for the three specific isoforms of the PRLR is expressed in both normal and cancerous human breast cells and tissues.

Ductal and lobular carcinomas are the most common histological types of breast cancer. This nomenclature and system of classification is not without controversy since both originate from the same anatomical structure, the terminal ductal lobular unit. Most pathologists label tumors by their grade, size, stage, and hormone receptor (estrogen receptor, ER; progesterone receptor, PR and Her2) status. Lobular carcinomas represent approximately 10% of breast cancers and are biologically distinct from ductal carcinomas [13] that have defined tumor foci. Lobular carcinomas appear spindly, tend to grow in sheets and, therefore, do not present as a mass. As a result, lobular carcinomas are more difficult to diagnose clinically and tend to be treated more aggressively [13]. But in spite of this, lobular carcinomas can be treated successfully by surgical or chemotherapeutic intervention. While there appears to be no survival advantage between the two types of cancers, development and progression of the disease varies [14,15].

On a molecular level, there are many differences between ductal and lobular carcinomas. Using microarray techniques and three types of statistical analyses, Zhao et al. [16] demonstrated that genes differentially expressed between ductal and lobular carcinomas code for proteins involved in cell motility/adhesion, fatty acid transport and metabolism, immune response, and electron transport. Most genes that significantly distinguish lobular carcinoma were involved in cell growth and immune response, but their function remains unknown.

Previous work using B6.2, a PRLR monoclonal antibody characterized in our laboratory [17] that is unable to distinguish the various isoforms, indicated a lack of correlation between PRLR expression and tumor grade, size or axillary lymph node status [18]. However, distinct differences were observed for the site of PRLR expression among normal, benign, and malignant breast tissue. Previous studies had suggested that in some subgroups of breast cancer patients, detection of PRLR may have prognostic significance [19]. With the discovery of the various isoforms of the PRLR, a more detailed analysis of the cellular localization of the receptor as well as possible differences between subtypes of breast cancer was warranted. To facilitate these studies we developed and characterized PRLR isoform specific polyclonal antibodies that reveal that three isoforms, LF, SF1a and SF1b, are differentially expressed in ductal and lobular carcinoma tissues.

## Methods

### Preparation of the polyclonal isoform specific antibodies

Synthetic peptides were designed based on the regions of unique intracellular sequences of the PRLR splice variants (Table 1), synthesized (AnaSpec, Inc. San Jose, CA) by the solid-phase method and conjugated to Keyhole limpet hemocyanin [20]. New Zealand white female rabbits (3-5 kg) were immunized (Animal Pharm Services, Healdsburg, CA) with the conjugated peptides as previously described [20]. Crude antisera was covalently linked to Affi-Gel 15 (Bio-Rad, Richmond, CA) and affinity purified by anion-exchange chromatography as described [21].

**Table 1:** Peptide sequence

LF	259	KGFDAHLLEKGKSEELLSALGCQDFPPTSDYEDLLVEYLEVD
SF1a	259	KGFDAHLLEKGKSEELLSALGCQDFPPTSDYEDLLVEYLEVD
SF1b	259	KGYSMVTCIFPPVPGPKI**KGFDAHLLEVTP**
LF	319	DSEDQHLMSVHSKEHPSQGMKPTYLDPDTDSGRGSCDSPSL
SF1a	319	DSEDQHLMSVHSKEHPSQGDPLMLGA**SHYKNLKSYRPRKIS**
LF	360	LSEKCEEPQANPSTFYDPEVIEKPENPETTHTWDPQCIS
SF1a	360	SQGRLAVFTKATLTTVQ
LF	400	MEGKIPYFHAGGSKCSTWPLPQPSQHNPRSSYHNITDVCELAVGPA
	445	GAPATLLNEAGKDALKSSQTIKSREEGKATQQREVESFHSETDQD
	490	TPWLLPQEKTPFGSAKPLDYVEIHKVNKDGALSLLPKQRENSGK
	535	PKKPGTPENNKEYAKVSGVMDNNILVLVPDPHAKNVACFEESAKEA
	580	PPSLEONOAEKALANFTATSSKCRLOL**GGLDYLDPACFTHSFH**

**bold **indicates unique synthesized sequences

### Cell culture and transfection

Chinese hamster ovary cells (CHO-K1, ATCC, Manassas, VA) were maintained in a-MEM (Invitrogen, Gaithersburg, MD) supplemented with 5% fetal bovine serum (FBS, Invitrogen) and penicillin/streptomycin (100 U/ml and 100 ug/ml respectively, Invitrogen). Transfections were performed using FuGENE 6 (Roche Applied Science, Indianapolis, IN) at a ratio of 1 μg DNA to 3 μl FuGENE. The PRLR isoform specific cDNA constructs were previously described [7]. Cells were transfected for 48 hr, then allowed to grow for an additional 48 hr.

T47D and MDA-MB-231 cells (purchased from ATCC, Rockville, MD) were grown in RPMI1640 supplemented with 5% FBS, penicillin (100 U/ml), streptomycin (100 μg/ml) and 10 μg/ml insulin. MCF7 cells (ATCC) were routinely grown in DMEM supplemented with 5% FBS, penicillin/streptomycin, and insulin. All cells were maintained at 37° in a humidified chamber under 5% CO_2 _in air.

### Western blot analysis

Transfected CHO cells were collected and whole cell lysates were prepared in Complete Buffer (Roche Applied Science) according to the manufacturer's instructions. Total protein was estimated according to Bradford [22]. Protein (100 μg) was subjected to 10-20% SDS-PAGE (Invitrogen). Proteins were transferred to nitrocellulose membrane and probed with isoform specific antibodies. Commercially prepared antibodies to the extracellular domain or SF1a of the PRLR (Invitrogen) were tested in parallel. Lysates from MDA MB 231 or MCF7 xenografts were similarly analyzed.

For immunoprecipitation studies, 500 μg of protein from whole cell lysates was bound to 10 μg/ml of isoform specific antibody [7]. Equal amounts of protein (100 μg) were separated by 10-20% SDS-PAGE and probed with its respective antibody. Reactivity was detected using ECL Plus (GE Healthcare Life Science, Pittsburgh, PA). Molecular size determinations were made using BenchMark Protein Ladder (Invitrogen).

### Tumor xenografts in mice

Female athymic nude mice (4-6 wks of age) were purchased from the NCI colony (APA, Frederick, MD). All animals were maintained on a 12 hr light/12 hr dark schedule with free access to laboratory chow and water. All animal experiments were conducted in accord with accepted standards of humane animal care and approved by the Animal Care and Use Committee at the National Institutes of Health. Breast cancer cells (2 × 10^6 ^cells) were injected into the cleared mammary fat pads [23] and monitored for tumor formation. Once tumors reached 1 cm^2 ^(measured in two dimensions, length × width), they were excised and fixed in 10% normal buffered formalin (Fisher, Pittsburgh, PA). Four micron thick sections were cut and stained with hematoxylin/eosin for histological examination or used for immunohistochemistry.

### Immunohistochemistry

Immunostaining for specific PRLR isoforms (10 μg/ml) was carried out using the Vectastain ABC kit (Vector Laboratories, Burlingame, CA) according to the manufacturer's instruction. Color was developed with diaminobenzidine peroxidase (DAB) substrate kit (Vector) and counterstained with hematoxylin.

### Fluorescent immunocytochemistry

CHO cells were plated on 8-well glass chamber slides (Nunc, Rochester, NY) and transfected as above. After blocking in 5% normal goat serum (Jackson Laboratories, Bar Harbor, ME) prepared in PBS-0.1% Triton, slides were incubated with the PRLR isoform specific antibodies (10 μg/ml) for 2 hr at room temperature. In all cases no primary antibody served as the negative control. Slides were washed four times with PBS-0.1% Triton followed by incubation for 1 hr with red fluorescent tagged goat anti-rabbit secondary antibody (AlexaFluor 594, 1:500, Invitrogen) in the dark. After extensive washing with PBS containing Triton, slides were mounted with Prolong Gold antifade reagent with DAPI (Invitrogen). The fluorescent staining pattern of the receptor isoforms was evaluated using an Olympus BX40 fluorescence microscope (Olympus America, Center Valley, PA).

For fluorescent immunohistochemistry breast tumor samples were supplied by either the Cooperative Human Tissue Network, a NCI supported resource that supplies human biospecimens to IRB approved researchers or as high density breast arrays purchased from US Biomax, Inc (Rockville, MD). The tissues obtained for analysis were considered pathological medical waste; thus any clinical details of the women were unattainable. In addition, the specimens were fixed in formalin to most closely replicate tissue processing in the clinic. PRLR isoform expression was examined on 12 lobular carcinoma and 10 ductal carcinoma specimens obtained from CHTN; other investigators may have received samples from these same tissues. Samples were fixed in 10% normal buffered formalin, embedded, cut into four micron sections, and deparaffinized prior to staining. Two separate tissue arrays, one containing 188 individual cases consisting of multiple types of infiltrating ductal (144), lobular carcinoma (24), and normal breast and the other containing 80 individual lobular carcinomas were utilized; 10 cores from the latter array were used as negative controls (no primary antibody). Slides were deparaffinized and antigen retrieval was performed according to the manufacturer's recommendations. Sections were permeabilized in PBS-0.1% Triton for 5 min and stained as above.

### Measurement of fluorescence intensity

Because serial sections for the tumor samples and on the tissue arrays were used, the same region of each tissue could be measured for fluorescence intensity using Adobe Photoshop (Adobe Systems Inc., Beaverton, OR). Nearly every cell in positive samples showed some level of PRLR isoform expression; as a result, red fluorescence intensity was used to compare levels of isoform expression between samples. In order to do this, the same fluorescent areas were selected from each serial section using the lasso and rectangular marquee tools. Selected sections were analyzed using the histogram function through the red channel, which gave the mean red intensity of the selected section. Photoshop assigns intensity values between 0 and 255 to each pixel in the selected area and then averages these intensities. The distribution of these means was analyzed and used to arbitrarily divide samples into four intensity classes: negative (less than 30 intensity), low (between 30 and 50 intensity), medium (between 51 and 70 intensity), and high (greater than 70 intensity).

### Total RNA isolation and quantitative real-time PCR

Total RNA was isolated using TRIzol reagent (Invitrogen) according to the manufacturer's instructions. The SuperScript III Reverse Transcription kit (Invitrogen) was used to reverse transcribe 1 μg of total RNA in a total volume of 25 μl. The real-time PCR reaction was performed using the Stratagene Brilliant II SYBR green QPCR Kit (La Jolla, CA) as suggested using 10 pmol of forward and reverse primers for each PRLR isoform [24] in a Stratagene Mx3005P starting with a 10 min incubation at 95°C followed by 40 cycles (95°C for 30 sec, 55°C for 1 min, 72°C for 1 min). Data were analyzed using the ΔΔC_T _method (Livak and Schmittgen 2001) with GAPDH as the housekeeping control.

## Results

### Characterization of isoform specific antibodies

**Figure 1:**
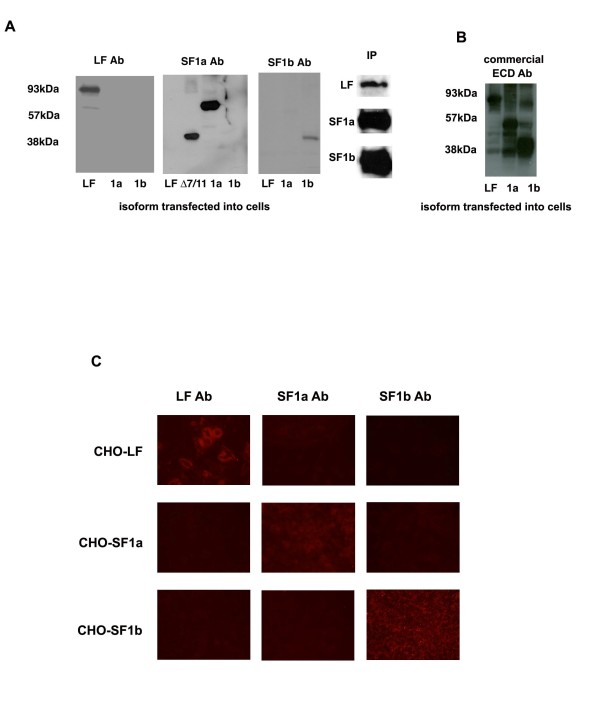
**Characterization of PRLR isoform antibodies. **A. Western blot analysis indicating specificity of polyclonal antibodies. CHO cells were transiently transfected with isoform specific PRLR cDNA as described. Cell lysates were prepared and proteins separated by PAGE. Each isoform specific lysate was probed with each isoform specific antibody. Each transfection was performed in triplicate and Western blot analysis performed twice. Data shown are a representative autoradiogram. Molecular weights are marked as indicated: LF = 93 kDa, SF1a = 57 kDa, SF1b = 38 kDa. Cell lysates from CHO transfected cells were immunoprecipitated with their respective antibodies, then immunoblotted with the same antibodies. Each transfection was performed in triplicate and Western blot analysis performed twice. Data shown are a representative autoradiogram. IP indicates immunoprecipitation. B. Western blot analysis using commercially available PRLR antibody. ECD = antibody recognizing extracellular domain C. Fluorescent immunocytochemical analysis. CHO cells were transiently transfected with isoform specific PRLR cDNA as described. Specific staining was observed using isoform specific polyclonal antibodies. The negative control (not shown) lacks primary antibody and appears black. Data shown are representative of triplicate experiments. Magnification 20×.

By RT-PCR, we had previously demonstrated that PRLR was overexpressed in 80% of biopsy samples compared to normal tissue [7]. However, until now, there were no reliable antibodies available to examine the PRLR isoform profile by immunohistochemical techniques. To characterize PRLR expression in human breast cancer samples requires robust antibodies that are specific to each isoform. Thus we prepared and characterized polyclonal antibodies that specifically identify each of the major cell associated isoforms, LF, SF1a and SF1b. CHO cells were transiently transfected with cDNA containing the individual PRLR isoforms; Figure. 1A shows the specificity of our antibodies by western blot. Cell lysate was separated by SDS-PAGE and probed with the antibodies. Each antibody correctly bound to its corresponding protein. No cross-reactivity was seen. The antibody prepared against SF1a isoform was also able to bind the PRLR isoform Δ7/11 as it shares sequence homology with SF1a but lacks the transmembrane domain (exons 7-11, [7]). Using our polyclonal antibodies in immunoprecipitation experiments enhanced the intensity of signal produced by western blot analysis, but did not change the overall results (Figure 1A, far right).

In parallel, two commercially available antibodies were also tested by western blot. A monoclonal antibody (Invitrogen, 95-9200), raised against the extracellular domain common to all PRLR isoforms, recognized all three isoforms from transiently transfected CHO cells (Figure 1B) as did the polyclonal antibody (Invitrogen, 34-9600) against SF1a (data not shown).

In addition, by immunocytochemical analysis, we were able to stain CHO cells transiently transfected with PRLR isoform specific cDNA (Figure 1C). Only cells expressing the individual isoforms showed positive staining with its respective antibody. No cross-reactivity was identified using the antibodies on cells transfected with non-corresponding PRLR cDNA. Thus, each PRLR antibody was able to specifically detect its own isoform by both western blot and immunocytochemical analyses.

### Immunostaining of human breast cancer xenografts

**Figure 2:**
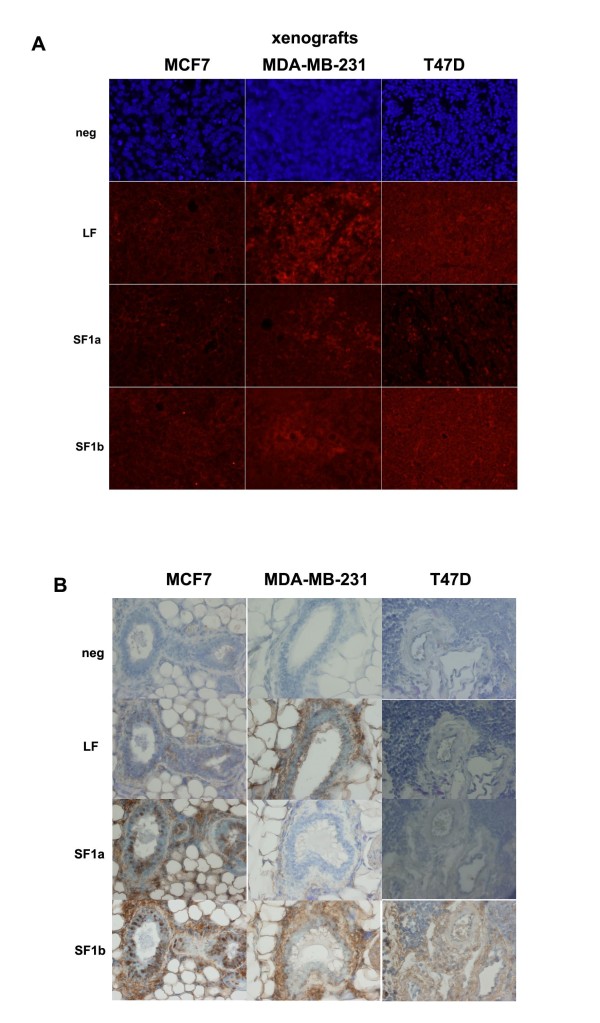
**Immunoanalysis on human xenografts. **A. Fluorescent immunohistochemistry was performed on MCF7, MDA-MB-231, or T47D cells which were injected into the cleared fat pads of nude mice; once tumors reached 1 cm^2 ^(measured in two dimensions), they were excised, formalin fixed, sectioned, and stained with the indicated PRLR isoform specific antibodies. B. Immunohistochemical analysis using DAB as chromogen of MCF7, MDA-MB-231, or T47D xenograft tumors. neg = no primary antibody as negative control; DAPI stain to visualize the presence of cells. Photographs are representative from 5 different tumors for each cell line. Magnification 40×.

Once we fully identified the specificity of the polyclonal antibodies using transiently transfected CHO cells, we tested their utility on xenografts obtained from human breast cancer cell lines. MCF7, MDA-MB-231, or T47D cells were injected into the cleared mammary fat pads of nude mice to produce xenografts. As expected, based on our previous studies examining the mRNA levels of the PRLR isoforms by RT-PCR [7], the immunofluorescent staining also showed considerable variation in PRLR protein expression among the 3 groups of xenografts examined (Figure 2A). LF antigen was expressed in all the xenografts to varying degrees. SF1b was highly stained in xenografts from all three cell lines. SF1a was comparably less visible in MCF7 and MDB-MB-231 xenografts, but was also expressed at low levels in sections from T47D xenografts. Similar results were observed using DAB as the chromogen (Figure 2B), a detection method that is used most commonly in the clinics, with one exception. LF expression was less visible in MCF7 and T47D xenografts with DAB when compared to immunofluorescent staining. This difference may be due to how the antibody binds to either horseradish peroxidase (for DAB) or FITC (for fluorescence) conjugated secondary antibody.

**Figure 3:**
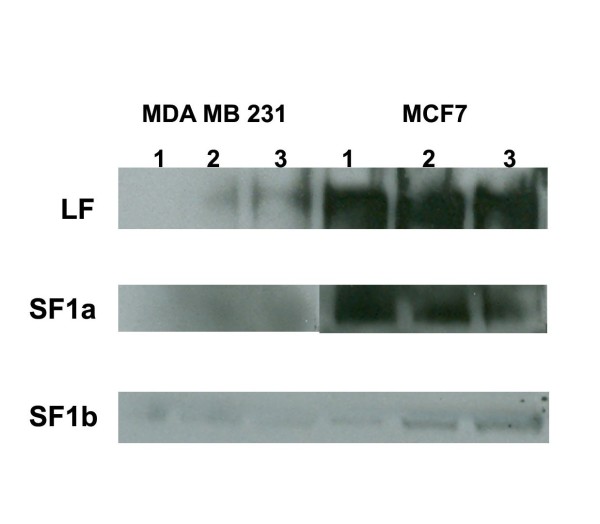
**Western blot analysis using lysates prepared from MDA MB 231 or MCF7 xenografts. **Three separate tumors from three separate animals for each cell type were separated by PAGE, probed with either LF, SF1a, or SF1b specific antibody, and detected by ECL.

Differences in PRLR isoform expression was also confirmed by western blot analysis using xenograft lysates prepared from tumors arising from MDA-MB-231 or MCF7 cells (Figure 3). Relative levels of expression varied between immunohistochemical and western analyses, possibly due to differences in the expression of the exposed epitopes. All three isoforms were detected in all the tumors, with LF and SF1a more highly expressed than SF1b in MCF7 xenograft lysates. Expression of the isoforms was less apparent, but still observed, in lysates from MDA-MB-231 xenografts. These data suggested that the isoforms of the PRLR are found in human breast cancer and suggest that the distribution of the PRLR isoforms could be used to qualitatively distinguish subtypes of breast cancer.

### PRLR isoform gene expression by qPCR

**Figure 4:**
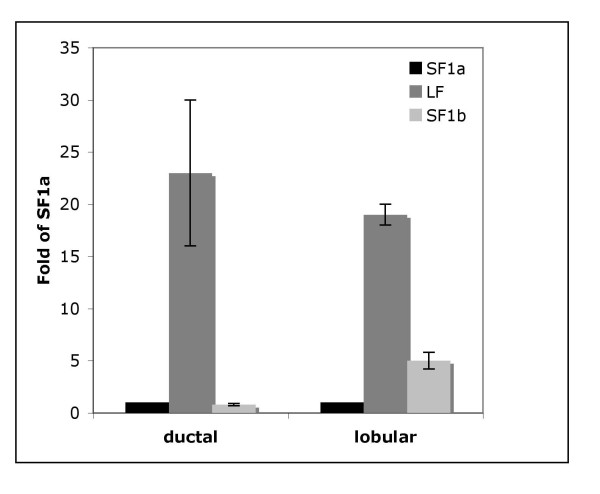
**Message for PRLR isoforms in breast carcinomas. **RNA was extracted from 7 ductal (left) and 7 lobular (right) carcinomas and qPCR was performed. Data shown are the mean +/- SE of delta delta Ct values relative to SF1a expression.

To characterize PRLR isoforms in breast cancer we performed qPCR (Figure 4) on 7 ductal and 7 lobular carcinoma specimens selected at random from samples that were also immunostained using our isoform specific antibodies. Samples were obtained from the CHTN as pathological waste with no clinical data. Message levels for SF1a was very low for all samples tested; as a result, message for LF or SF1b was compared to SF1a. As depicted in Figure 4, mRNA levels for LF were comparable between ductal and lobular carcinoma biopsies. While it was difficult to specifically quantify SF isoform expression by qPCR, in the lobular carcinoma samples analyzed, SF1b expression was nearly 2-fold higher than that in ductal carcinomas. These data suggested that isoforms expression could be used to distinguish breast cancer subtypes. Since we had previously demonstrated that genotype may not always correlate with phenotype [25], we examined the staining pattern in 12 ductal and 10 lobular carcinomas (data not shown). Similar to the staining pattern identified in xenografts, we were able to observe wide differences in the staining intensity across the examined samples. The amount of PRLR isoform expression is as varied as the individual necessitating analysis of larger numbers of samples.

### PRLR isoform expression in human breast biopsy samples

**Figure 5:**
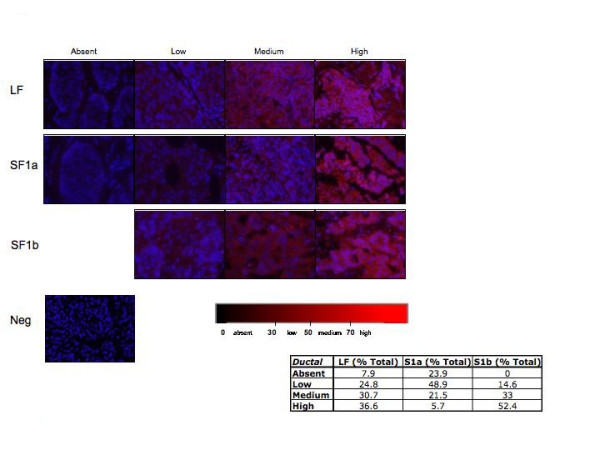
**Immunohistochemical analysis from ductal breast tissue arrays. **Representative photographs taken of ductal carcinoma specimens to demonstrate absent, low, medium, or high amounts of fluorescent red intensity staining. 144 individual ductal specimens were stained with PRLR isoform specific antibodies and analyzed. Scale bar indicates range of red scoring.

**Figure 6:**
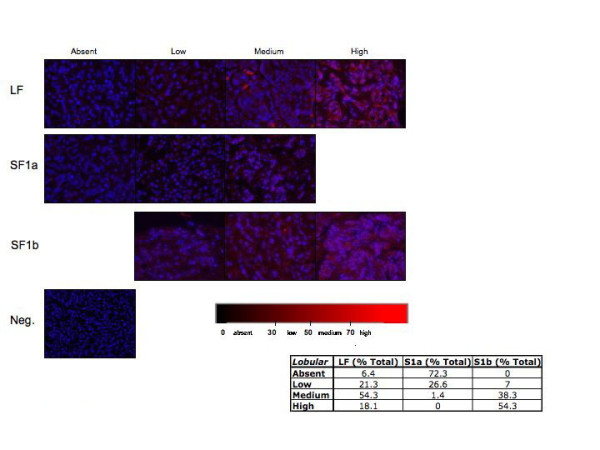
**Immunohistochemical analysis from lobular breast tissue arrays. **Representative photographs of lobular carcinoma specimens to demonstrate absent, low, medium, or high amounts of fluorescent red intensity staining. 104 individual lobular specimens were stained with PRLR isoform specific antibodies and analyzed. Scale bar indicates range of red scoring.

**Figure 7:**
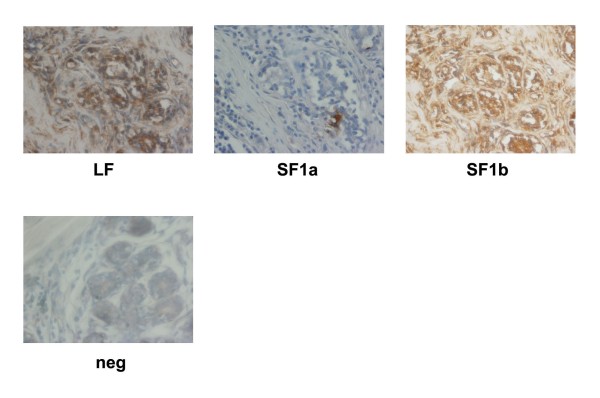
**Immunohistochemical analysis of a representative ductal carcinoma using DAB as a chromogen. **neg = no primary antibody as negative control; DAPI stain to visualize the presence of cells. Magnification 40×.

In order to determine whether the PRLR isoform expression pattern could aid in identification of breast cancer subtype, we examined 144 ductal and 104 lobular carcinoma biopsies available on tissue arrays with limited clinical information. Similar to what was observed in the individual tumor section above, staining for both the ductal and lobular carcinoma biopsies present on the tissue arrays varied (Figures 5 and 6). The vast majority of the samples, regardless of whether they were of ductal or lobular origin, stained for PRLR LF (ductal = 92%, lobular = 94%; see inserts Figures 5 and 6). However, ductal carcinoma specimens showed a nearly 2-fold higher percentage of high LF staining when compared to lobular carcinomas. In addition, differences were observed when staining for PRLR short forms. Staining with DAB as the chromogen also showed differences in PRLR isoform expression (Figure 7). While the distribution of expression levels of SF1b were similar for ductal and lobular carcinomas, the majority (76%) of ductal carcinoma biopsies expressed SF1a while the majority of lobular carcinomas lacked SF1a staining (72%) and 27% had only low levels of expression. These data suggest that the relative expression of levels of PRLR isoforms may aid in diagnosis of ductal vs. lobular carcinomas. Obviously more samples, with attendant clinical information, would need to be examined for statistical validation and to determine whether PRLR SF expression could be routinely used to distinguish between the breast cancer subtypes.

## Discussion

Recent evidence indicates that high levels of PRL in blood imparts increased risk of developing breast cancer, independent of other risk factors [2]. While analysis of the case-controlled prospective Nurses' Health Study cohort of 851 patients found no difference in relative risk for postmenopausal breast cancer in ductal vs lobular cancer with elevated PRL plasma levels [26], our more recent data using a population-based case-controlled study of 773 women from Poland has shown that serum PRL levels were significantly higher in post-menopausal women with invasive lobular compared to ductal carcinoma [27].

The incidence of ductal carcinomas has remained steady from 1987-1999 while the rate of lobular carcinomas has increased [28]. Perhaps this change is due to better detection methods, even though lobular carcinoma is generally more difficult to diagnose. Work done by Zhao et al. [2,4,16] has shown that, at the molecular level, lobular carcinomas have distinct gene expression patterns compared to ductal carcinomas. Whether these differences suffice to choose treatment options is open for debate. It is becoming clear that the future of cancer care will rely on personalized medicine. There are multiple types or classifications of breast cancer and in order to more rationally deliver the most effective treatment we must know as much as possible about an individual patient's disease at the molecular level. This includes all systems that impact on the cell fate of precancerous and cancerous breast cells. Breast tumors are currently classified by the expression of ER and PR and whether they overexpress Her2. These receptors are used because of their known involvement in development and progression of breast cancer. Recent evidence has clearly indicated that PRL plays a role in human breast cancer [2,4] and hence the receptors for this important hormone should also be assessed in all breast cancer cases.

PRL's action is mediated by its receptors that exist in multiple isoforms with common extracellular ligand binding and transmembrane domains but different intracellular domains resulting from alternate splicing [7]. Three major cell-associated isoforms are the LF, and SF1a and SF1b. While the functions and signaling pathways utilized by the LF have been studied extensively [4], similar studies of the short forms have been sparse. While it has been known for some time that the short forms of the PRLR can act as dominant negatives of the LF in transfected cells either by heterodimerization [7,11,29] or down regulation of expression of the LF [8,30], their function in the native setting has not been determined. In transfected cells, SF1a is a weak dominant negative of the LF for differentiation [7]. However, SF1a, as a homodimer, has the ability to activate the casein promoter [11,31]. SF1b is a strong dominant negative of the differentiative function of LF. In prostate cancer, treatment with the PRL inhibitor S179D PRL upregulated the expression of SF1b thus leading to upregulation of p21, a cell cycle inhibiting protein, and the vitamin D receptor known to promote differentiation [32]. Long-term, increased expression of SF1b not only decreased growth of prostate cancer cells in culture but also decreased cell migration and enhanced cell-matrix interactions and cell-cell aggregation [33]. Recent evidence has shown that the PRLR was overexpressed in ductal vs acinar adenocarcinoma of the prostate at both the transcript and the protein level [34]. PRLR transcripts identified from microdissected cell populations were elevated 6-fold in ductal vs. acinar carcinoma cells. Validation by immunohistochemical analysis, using an antibody that does not distinguish the isoforms, showed diffuse strong staining in 75% of ductal carcinoma regions in the 20 mixed acinar-ductal adenocarcinoma cases compared to 20% in acinar carcinoma regions. The majority of the acinar carcinoma regions showed no staining or low levels of patchy staining. That SF1b can inhibit the expression levels of LF through accelerated degradation of the LF message [30] suggests that the LF:SF1b ratio may be relevant to tumor growth.

PRLR transcripts have been described in up to 90% of breast cancers suggesting that their presence or absence may not be as important as the distribution of the isoforms. It is clear that mRNA for all three forms are present in many breast cancers [7,12]; the distribution of the isoform protein has not been ascertained. To properly assess this, isoform specific antibodies needed to be developed [35]. The polyclonal antibodies described in this paper provide the first clearly isoform specific tools that can be used to determine where and how the isoforms interact.

## Conclusions

We have successfully developed PRLR isoform specific antibodies that can be used to detect proteins by western blot, immunoprecipitation, and immunocyto/histochemical analyses. These antibodies may help us correlate receptor isoforms in both breast development and in cancer. We found differences in expression levels for the PRLR isoforms in lobular vs. ductal carcinomas that demonstrate these antibodies may be used as a new clinical tool to distinguish between subclasses of breast cancer. These insights may prove important in the treatment of breast cancer and other PRL responsive diseases.

## List of abbreviations

PRL: prolactin; PRLR: prolactin receptor; PRLR-LF: prolactin receptor long form; PRLR-SF1a or -SF1b: prolactin receptor short form 1a or 1b.

## Competing interests

The authors declare that they have no competing interests.

## Authors' contributions

EG designed experiments and wrote the manuscript. EG, SA, SL, ST, LJ, and LP conducted the experiments. PG designed the peptides and purified the antibodies. CDH tested the antibodies by western blot. BKV developed the ideas, helped design experiments and edited the manuscript. All authors have read and approved the final manuscript.

## Pre-publication history

The pre-publication history for this paper can be accessed here:

http://www.biomedcentral.com/1471-2407/10/678/prepub
